# Detection of Maternal and Cytoplasmic Effects on Resistance to *Zymoseptoria tritici* in Durum Wheat

**DOI:** 10.1155/2022/8497417

**Published:** 2022-03-29

**Authors:** Marwa Hassine, Fethi Bnejdi, Bochra Amina Bahri, Salma Tissaoui, Amira Mougou-Hamdane, Mouna Guesmi, Mokhtar Baraket, Hajer Slim-Amara

**Affiliations:** ^1^LR14AGR01, Laboratory of Genetics and Cereal Breeding, National Agronomic Institute of Tunisia, University of Carthage, Tunis 1082, Tunisia; ^2^Laboratory of Biodiversity, Biotechnology and Climate changes, Faculty of Sciences of Tunis, University of Tunis El Manar, 2092, El Manar II, Tunis, Tunisia; ^3^The Higher Institute of Agronomic Sciences of ChottMariem, University of Sousse, Sousse, Tunisia; ^4^Department of Plant Pathology and Institute of Plant Breeding, Genetics, And Genomics, 228 Turfgrass Research & Education Center, University of Georgia, Griffin, GA 30223, USA; ^5^LR14AGR02, Laboratory of Bio-aggressors and Integrated Pest Management in Agriculture, National Agronomic Institute of Tunisia, University of Carthage, Tunis 1082, Tunisia; ^6^Regional Field Crops Research Center Beja, Tunisia; ^7^University of Carthage, National Research Institute of Rural Engineering, Water and Forestry, Rue Hédi EL Karray El Menzah IV 1004 2080 Ariana, Tunisia

## Abstract

Septoria tritici blotch (STB) is a major disease problem of wheat worldwide. To optimize the introgression of resistance genes in elite genotypes throughout traditional or molecular breeding programs, a full understanding of the quantitative inheritance of resistance to *Zymoseptoria tritici*, plant height (PH), and thousand kernel weight (TKW) is needed. In this study, maternal and cytoplasmic effects of resistance to STB were investigated using P1 (susceptible, high-yielding line) and P2 (resistant, low-yielding line) durum wheat lines and their F_1_, RF_1_, F_2_, RF_2_, BC_1_, RBC_1_, BC_2_, and RBC_2_ progeny, assessed for resistance to STB during three growing seasons. Duncan mean's analysis revealed significant differences between generation means for STB, PH, and TKW. The two parents had an extreme pattern. The F_1_ and RF_1_ segregated close to their respective parents, suggesting the presence of cytoplasmic and maternal genetic effects for *Z. tritici* resistance, PH, and TKW. Separate generation mean's analysis confirmed the results of the Duncan test. A three-parameter model was found to be not adequate for all traits in all three growing years; while a digenic epistatic model with cytoplasmic or/and maternal effect was adequate for all cases. Narrow-sense heritability was in the range of 50–60%, 30–69%, and 28–31% for STB, PH, and TKW, respectively. For STB, high heritability and the presence of fixable epistatic effect is encouraging and could lead to creating varieties with the right female parent to exploit cytoplasmic and maternal effects in order to improve resistance to *Z. tritici* in durum wheat.

## 1. Introduction

Septoria tritici blotch (STB), caused by the ascomycete fungus *Zymoseptoria tritici* (teleomorph: *Mycosphaerella graminicola* (Fuckel) Schroeter) [1], is the most important damaging pathogen of wheat worldwide [2, 3]. Particularly in Mediterranean regions [4], such as Tunisia, the serious and frequent epidemics reduce yields by over 50% mainly on *Triticum turgidum* L. var. *durum* Desf.) due to the important adaptation of *M. graminicola* strains to durum wheat varieties [5, 6]. Moreover, modern and improved varieties showed decreased yield due to STB resistance break down following monoculture practices [7] and due to the effect of climate change and unpredictable rainfall. These conditions greatly influence the severity of *Z. tritici* [8, 9] and the prevalence of sexual recombination of the pathogen [10, 11]. Therefore, STB epidemics in durum wheat in Tunisia during the growing season have been recurrent with the development of new resistant strains [12, 13]. In addition, the use of conservation agriculture that allows for the oversummering of this pathogen contributes to the conservation of highly virulent pathotypes [14]. The high levels of genetic diversity in STB populations [15] were responsible to the complexity of disease control, which still relies heavily on fungicides [3]. Extensive use of fungicides has led to numerous cases of resistance in *Z. tritici* [16, 17] to azole [18, 19] and strobilurin fungicides [20, 21] and insensitivity to succinate dehydrogenase inhibitors (SDHIs) [22] and to environmental pollution [23]. This has made Septoria resistance one of the highest priorities in wheat breeding programs as well as research on effective, economic, and environmentally safe alternatives to reduce yield losses [3, 24]. Therefore, a continuous effort has focused on host resistance which could play the lead role in increasing the durability and effectiveness of the resistance genes in the commercial life time of released cultivars [25, 26]. Hence, this effective approach to disease control has led to the identification of 21 major qualitative *Stb* genes and 167 quantitative trait locus in wheat [24, 27]. However, the qualitative resistance is effective only against the virulent genotypes of *Z. tritici*, and resistance can be overcome through the evolution of pathogen virulence [28, 29]. In contrast, quantitative resistance is polygenic and provides partial resistance to a wide variety of isolates in order to provide a durable resistance under field conditions [30–32]. The inheritance of this resistance may follow dominant, partially dominant, epistatic, recessive, additive, and nonadditive gene action [33, 34]. Only a few studies have reported the absence of an epistatic effect and the presence of an additive effect in inheritance of resistance to STB in durum wheat [7]. On the other hand, several reports have focused only on nuclear gene effects and found that inheritance to *Z. tritici* was governed by additive, dominance, and epistatic effects [35–38]. Therefore, it is considered of great importance to identify the cytoplasmic genetic information as a source of genetic diversity and to evaluate the existence of reciprocal resistance effects to this pathogen [39, 40]. In this context, the objective of the present study was to investigate the contribution of maternal and cytoplasmic effects in inheritance of resistance to STB, in order to provide additional understanding of *Z. tritici* resistance in durum wheat.

## 2. Material and Methods

### 2.1. Plant Material and Population Development

**Figure 1:**
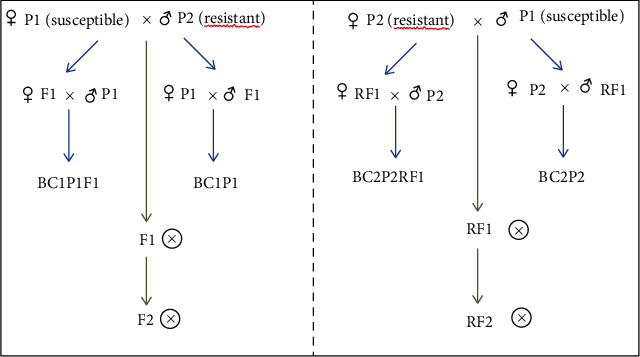
Crossing diagram adopted for the field trials in order to assess the cytoplasmic resistance through generations evaluated in 2015, 2016, and 2017.

In order to determine the presence of the cytoplasmic resistance, several populations were developed from the crosses between two Tunisian durum wheat varieties selected on the basis of their differential reaction to *Z. tritici*. The susceptible parent “Karim” (P1), highly appreciated by farmers for its good agronomic performance, yield stability, and industrial quality and which covers more than 60% of the durum wheat area in Tunisia, was crossed with the STB-resistant genotype “Maâli” (P2). The resistant parent Maâli was released in Tunisia in 2007 and showed good levels of resistance to STB over at least the last 8 years at different locations within the country (Gharbi, unpublished data). Hence, improving Karim resistance is highly sought by wheat breeders in response to farmer and industrial needs. These two parents were used as males providing nuclear inheritance and as females providing both nuclear and cytoplasmic inheritance in two different crosses. The F1and RF1of direct and reciprocal crosses were obtained by hand emasculation and pollination in the field at the Regional Fields Crop Research Center (CRRGCB) at Oued Beja (Beja Governorate, Tunisia) (36°44′05^″^N, 9°13′35^″^E) during the 2013–2014 growing season. These generations were self-pollinated to produce F2 and RF2, respectively. The backcrosses to both parents (resistant and susceptible) using the F1 plants as females or as males during 2014–2015 seasons were denoted BC_1_P_1_ and BC_2_P_2_. These different populations (P1, P2, BC1, RBC1, BC2, RBC2, F1, RF1, F2, and RF2) were evaluated under field conditions during the 2015, 2016, and 2017 growing seasons (Figure 1). The different progenies were tested at this experimental station which is characterized by a subhumid bioclimatic and an average annual rainfall of 500–850 mm with mild winter, which are favorable conditions for natural infection of Septoria. This area is particularly known to be a hotspot for STB in north-western Tunisia. A total of 1300 plants per year were evaluated for the disease in unreplicated field trials, across the three years (2015–2017) for each of the different populations and generations. The experiment used an alpha design [41]. Parents were used as controls, and each accession was sown as a single row of 1.5 m length spaced 25 cm apart at a rate of 40 seeds per meter [7]. Weeds were controlled at both pre- and postemergence with the appropriate herbicides applied at the seedling growth stage between first and second node stages [42]. During the three growing seasons, plots were fertilized (33.3 kg/ha of N) at tillering and stem elongation stages (Zadoks scale GS29 and GS69) [42].

### 2.2. Experimental Trials, Inoculum Preparation, and Plant Inoculation

During the first growing season (2015), experimental trials were under STB natural infection. During the 2016 and 2017 growing seasons, artificial inoculations with a mixture of Septoria isolates were performed by spraying a spore suspension using a CO_2_-pressurized knapsack sprayer with a 1 m hand-held boom till run-off. These inoculations were applied two times at the 21 and 37 Zadoks growth stages [25, 42], in order to initiate and ensure a high disease pressure. The inoculums used in this study were obtained from infected leaves of the susceptible durum wheat cultivar Karim randomly collected during the 2015 crop season and originating from the same field trials. The preculture of the inoculum was performed for 6–8 days on potato dextrose agar. A fresh piece of the pathogen colonies from agar plates was inoculated in an autoclaved 500 ml Erlenmeyer flask containing 250 ml of yeast-glucose liquid medium (30 g of glucose and 10 g of yeast per liter of demineralized water and autoclaved later on). These flasks were incubated in a rotary shaker at 100 rpm and 20°C for 7–10days [43]. The produced spore suspensions were collected after overnight settling in static cultures. The inoculum concentration was adjusted to 10^6^ spores/ml in a total volume of 50 ml and was supplemented with two drops of Tween 20 prior to inoculation [7, 31]. The different isolates used as mixtures in these experimental trials were previously tested on five predominant cultivated varieties (Karim, Nasr, Razzak, Maâli, and Salim) used in wheat breeding programs and known to have different levels of susceptibility to the Tunisian population of *Z. tritici*. The isolates were virulent on most of the tested durum wheat cultivars during the field trials (Table 1).

### 2.3. Disease Evaluation

The progeny from the two parents of each cross were evaluated for STB progress from the beginning of March until the end of May at four dates with 20-day intervals between each evaluation date, starting 21 days after the second inoculation [44]. These different stages were considered as a critical period for grain yield production because the reduction of the green leaf area on the flag leaf is responsible of the most significant yield losses [45]. The disease severity was evaluated using the double-digit scale (00–99) developed as a modification of the Saari-Prescott severity scale to assess wheat foliar diseases [46, 47]. The first digit (D1) indicates the relative height of the disease on the plant and corresponds to the vertical disease progression using the original 0–9 Saari-Prescott scale as a measure. At the level 0, the observations of disease symptoms are limited to the basal leaf of the plant, whereas at level 9, symptoms are present on leaves, sheaths, glumes, and barbs. The second digit (D2) represents the severity measured as coverage of leaf area with lesions bearing pycnidia on a scale from 0 (fully resistant) to 9 (fully susceptible) [41, 46, 48]. For global scoring of the disease severity, the percentage was calculated using the following formula (Sharma and Duveiller 2007; [31]):

(1) $$\begin{array}{c} \text{Severity}\left(\%\right)=\left(\text{D}1/9\right)\left(\text{D}2/9\right)\times 100. \end{array}$$

The area under disease progress curve (AUDPC) was subsequently used in the quantitative analyses of the temporal differences in Septoria progress [30, 49]. For each entry, the AUDPC was calculated [31, 50] as follows:

(2) $$\begin{array}{c} \text{AUDPC}=\overset{n-1}{\underset{i=1}{\sum }}\left(\frac{Yi+1+Yi}{2}\right)\left(ti+1-ti\right) \end{array}$$

where*Y*_*i*_ = the proportion of diseased plants at *i*^th^ observation, *t*_*i*_ = time of the *i*^th^ observation in days from the first observation, and *n* = total number of disease observations.

Three phenotypic classes for necrosis and pycnidia scores at the adult plant stage were used on the different generations (BC_1_, RBC_1_, BC_2_, RBC_2_, F_2_, and RF_2_) according to the qualitative scale of Rosielle [51] as slightly modified by McCartney et al.[34].

This scale is based essentially on the disease classification into three categories: S corresponds to the susceptible lines with severity responses similar to the susceptible parent Karim, R corresponds to the relatively resistant lines with severity responses similar to the resistant parent Maâli, and I corresponds to the lines segregating with intermediate response to STB. The R, I, and S genotypes displayed different necrosis and pycnidia ranges [25, 52, 53]. The 1–9 rating based on visual assessment of the top three leaf layers [54] was converted to S, R, and I as described by Berraies et al. [52]. The R genotypes have 0–25% (rating 1–4) of necroses and pycnidia; S genotypes have 75–100% (rating 7–9) of necroses and pycnidia; and I genotypes have 25–50% (rating 4–6) of necroses and pycnidia (Table 2).

The harvesting of the different generations was performed in June for each of the three growing seasons. The impact of the disease on two agronomic traits, PH and TKW, was studied. The PH was measured at the end of the vegetative plant cycle and represented the mean straw length from the seedling until the ear base of each shoot head [50, 55]. The TKW was determined for each of the 1300 plants by using a sensitive balance as described by Ben Mbarek and da Silva [50] and Alamirew et al. [56].

### 2.4. Statistical Analyses

Generation means' analysis was determined by joint scaling test as described by Rowe and Alexander [57], using the weighted least squares method [58–60]. The significance of each parameter was determined by *t*-test [61].

Homogeneity of variances of nonsegregating generation (P_1_, P_2_, and F_1_) was tested using Bartlett's test [62]. Additive, dominance, and environmental variance components were estimated using the maximum likelihood method with the observed variance of the six basic generations being used as the initial weights (df/2S^2^) until the chi-squared test value reached a minimum [59]. Narrow-sense heritability was calculated as follows: *h*^2^*n* = VA/VA + VD + VE, where VA is the additive genetic component of variance, VD is the dominance genetic component of variance, and VE is the environmental variance [58]. The dominance variance was negative and was set to zero.

The number of genes controlling resistance to STB was estimated as follows: *N* = (P1–P2)^2^[1.5–2 *h* (1–*h*)]/8[*σ*^2^F2–0.25 (*σ*^2^P1 + *σ*^2^P2 + 2*σ*^2^F1)], where *h* = (F1–P1)/(P2–P1).

To identify similarities between the different populations, principal component analysis (PCA) was performed using R package software version 4.0.5, basing on the mean data of PH, AUDPC, and TKW for each population. Results from PCA were displayed as a biplot to investigate the correlation between quantitative traits of the population responses across experiment.

## 3. Results

### 3.1. Means and Variances

**Figure 2:**
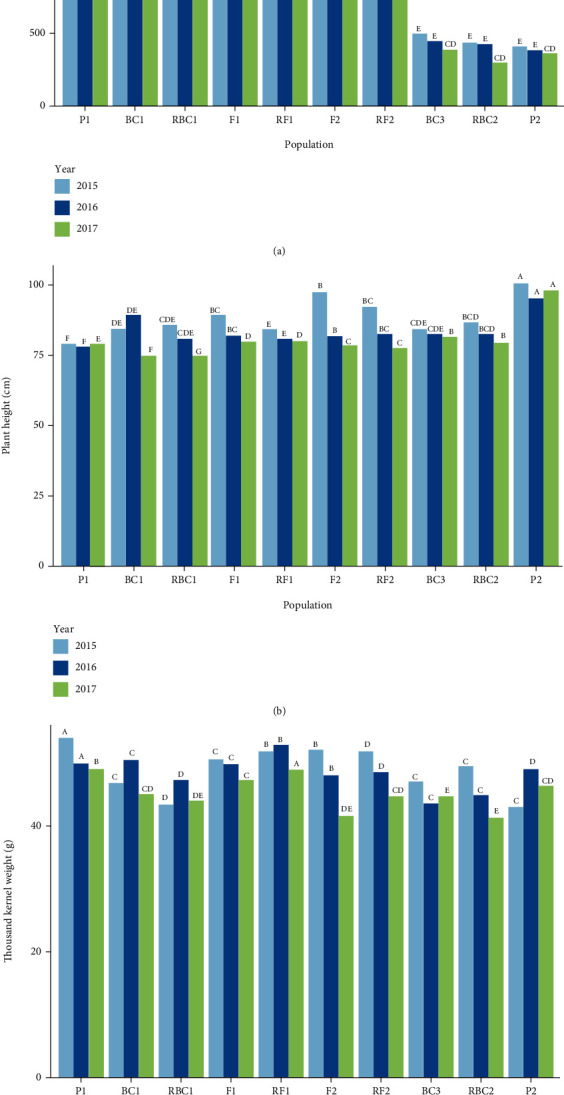
Evaluation of the area under disease progress curve (AUDPC) for resistance to *Zymoseptoria tritici* (a) (untransformed data), plant height (b), and the thousand kernel weight (c), in durum wheat crosses derived from the susceptible parent Karim (P1) and the resistant parent Maâli (P2) during three growing years. Means followed by different letters within each column and within each year significantly differed based on Duncan's test (*P* < 0.05).

The natural STB infestation slightly differed between all populations over the three growing seasons especially in the beginning of each season. During the crop seasons of 2015, 2016, and 2017, the Duncan means analysis revealed broad segregation for disease severity levels in all populations, which consisted of the two durum wheat parent lines and their F_1_, RF_1_, F_2_, RF_2_, BC_1_, RBC_1_, BC_2_, and RBC_2_ progeny. Both parental genotypes Karim and Maâli produced consistent susceptible and resistant reactions, respectively, to the pathogen in all infection phases under field conditions over the three-year period (Figure 2). The susceptible parental line (P1) showed more extreme disease severity compared to the other populations' means with a maximum value AUDPC of 2100 obtained in 2015, which was the most favorable year for the disease development. The F_1_ and RF_1_ populations means were situated between the resistant and susceptible parents and revealed higher average resistance over the three-year period. However, the AUDPC value of F_2_ and RF_2_ changed among the years and ranged from 1100 in 2017 to 1700 in 2015. This revealed that the average resistance of these populations was situated between those of the parents, and the RF_2_ mean was higher than that for F_2_. However, the means of BC_1_, RBC_1_, BC_2_, and RBC_2_ tended to be close to those their recurrent parent. The AUDPC indicated that the presence of cytoplasmic and maternal effects for the resistance to this disease were highly dependent on the female (resistant) parent Maâli.

The tested genotypes were also evaluated using the agronomic parameters PH and TKW, in order to estimate the impact of the severity of *Z. tritici* and the heritability of the different traits. The results emphasize that these two parameters were highly dependent on the impact of the disease in all different populations and on the three growing seasons (2015, 2016, and 2017). Moreover, and similarly to AUPDC, the genotypes closely related to the resistant parent (Maâli) had the greatest PH, range 88–110 cm across the different populations. Similarly, TKW was variable between the tested genotypes and RF_1_ and RF_2_ had averages close to that of P1 with 50 and 52 g, respectively. This TKW variability was expected due to the different resistance levels between the different populations over the years 2015, 2016, and 2017.

### 3.2. Gene Effects

The result of the three-parameter and the best-fit models is reported in Table 3. The chi-squared tests revealed that the additive-dominance model was not adequate. Therefore, the digenic epistatic model with epistasis effect was used and also appeared inadequate. The failure of the epistasis model may be due to the influence of the maternal effects governing inheritance of this trait. Consequently, a digenic epistatic model with maternal effect was applied and revealed to be adequate. The result of the best-fitted model indicated that for all three years (2015, 2016, and 2017), the additive effect on STB was negative and higher in magnitude than the dominance effect and ranged from −0.9 to −3.9 across 2015–2017. The additive × additive effect (I) was positive in all cases and varied from 2.7 to 3.4. However, the cytoplasmic effect (C) for AUDPC was negative in 2015 and positive in 2016 and 2017 and ranged from −0.4 to 0.92. The dominance × maternal (Hm) and the additive × maternal (Dm) effects were negative and varied, respectively, from 0 to 0.42 and from −6.9 to 0.5 during the three cropping seasons.

In addition, based on the three-parameter and the best-fit models, a negative additive effect for the TKW was revealed, ranging from −4.8 to −5.2. However, for SH, a positive additive effect was observed (12.7 and 7.42) in cropping seasons 2015 and 2017, respectively. In addition, the dominance effect was negative for TKW and was positive in one case and negative in the other case for SH (−4.21 to 7.9). However, only the additive × additive effect (I) and the cytoplasmic effect (C) were shown for these two agronomic parameters. A positive cytoplasmic effect (C) for PH was observed; while for TKW, a negative effect and a positive effect were observed in cropping seasons 2015 and 2017 with −0.6 and 1.6, respectively. This last variation from negative to positive effect was also observed for the additive × additive effect (I) for PH and TKW for the two growing seasons (2015 and 2017), respectively.

*a*, additive; *d*, dominance; *c*, cytoplasmic effect; am, additive maternal effect; dm, dominancematernal effect; *a* × *a*, additive × additive effect; *d* × *d*, dominance × dominance effect; *d* × *a*, dominance × additive effect; ^∗∗^significant at *P* < 0.01.

### 3.3. Narrow-Sense Heritability and Effective Factor

**Figure 3:**
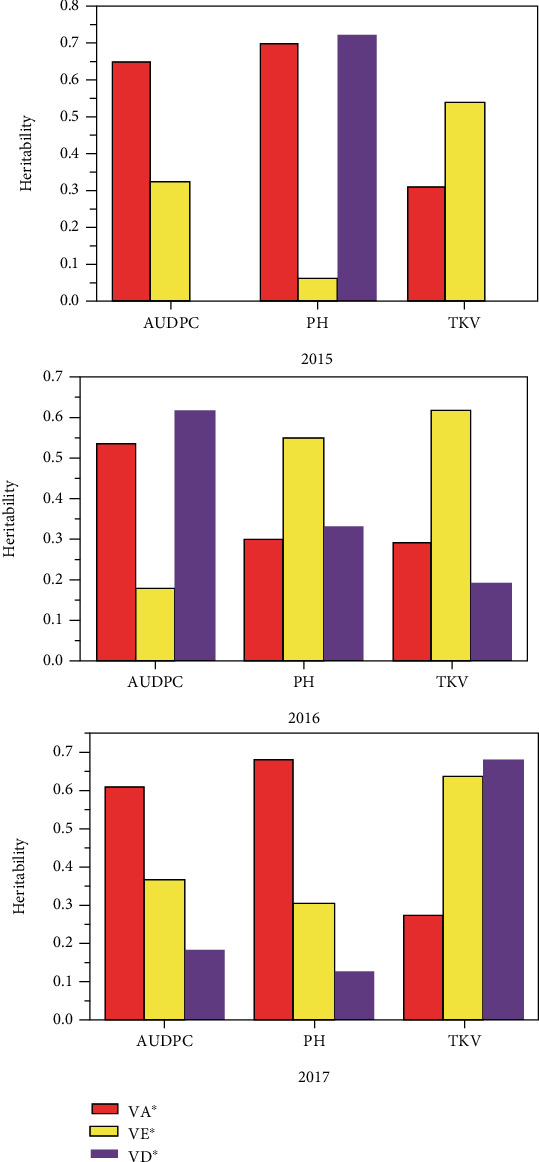
Estimation of heritability during three growing seasons (2015, 2016, and 2017) for the three factors studied: area under disease progression curve (AUDPC), the plant height (PH), and the thousand kernel weight (TKW) for all populations studied. VA: additive variance; VE: environmental variance; VD: dominance variance rate; where VA∗ = VA/VA + VD + VE, VD∗ = VD/VA + VD + VE, and VE∗ = VD/VA + VD + VE.

The variance components, narrow-sense heritability, and the effective factor are reported in Table 4 and Figure 3. The variance components were estimated and used to calculate narrow-sense heritability as described by Kearsey and Pooni [58]. The environmental variance was positive and varied between growing seasons from 0.1 (in 2015) to 0.3 (in 2017). The dominance for the resistance was positive with range 0.01–0.24. Moreover, the additive variance was positive and two-times higher than the environmental and dominance variances and with range 0.2–0.5%. The effective factor was estimated as described by Lande [63]. The minimum number of genes involved in resistance to *Z. tritici* varied depending upon the population and ranged from to 1.7 (2016) to 2.3 (2017).

**Figure 4:**
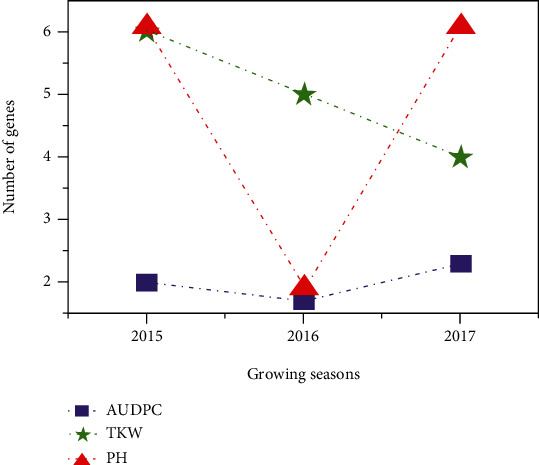
Number of genes involved in resistance to *Z. tritici* for the three traits studied: area under disease progression curve (AUDPC), plant height (PH), and thousand kernel weight (TKW) during three growing seasons for all populations.

Similarly, a positive environmental variance was observed across the three cropping seasons for the different generations tested for PH from 2.16 (2015) to 5.42 (2017) and for TKW from 3.5 (2017) to 7.23 (2015). In addition, the dominance and additive variances were positive across 2015, 2016, and 2017 seasons for these agronomic parameters. However, the minimum number of genes was in the range of 4–6 for TKW and 4–5 for PH (Figure 4).

PVC, population variance components; VE, environmental variance, VA^∗^, additive variance; VD^∗^, dominance variance; df, degrees of freedom, calculated as the number of generations minus the number of estimated variance parameters; ns, nonsignificant. *N* = (P1–P2)^2^ [1.5–2 *h* (1–*h*)]/8[*σ*^2^F2–0.25 (*σ*^2^P1 + *σ*^2^P2 + 2 *σ*^2^F1)], where *h* = (F1–P1)/(P2–P1) [63].

**Figure 5:**
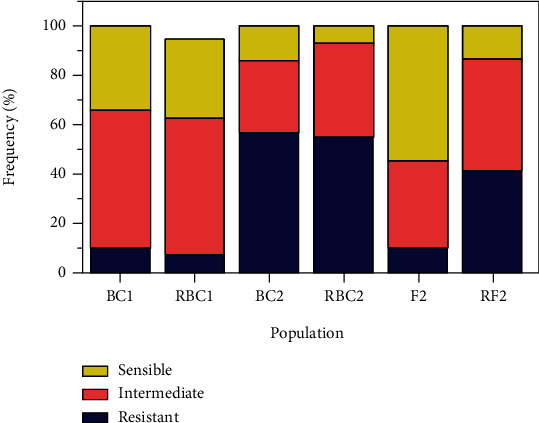
Frequency of resistant (R), intermediate (I), and susceptible (S) durum wheat populations tested for resistance to *Z. tritici* under field conditions during 2015, 2016, and 2017 cropping seasons.

The distribution of the resistance to STB in the different populations varied with the level of resistance of the female parent (Figure 4). This important observation enhanced the maternal effect on one side and the cytoplasmic impact on the other side on these different populations. The distribution of the frequencies (Figure 5) and the means for resistant traits were compared to the mid-parent value as described by Bnejdi et al. [61] and showed an important rate of resistant genotypes in the BC_2_, RBC_2_, and RF_2_ generations of 56.66%, 55.00%, and 41.00%, respectively. This distribution suggested that resistance was probably under polygenic control with additive loci.

**Figure 6:**
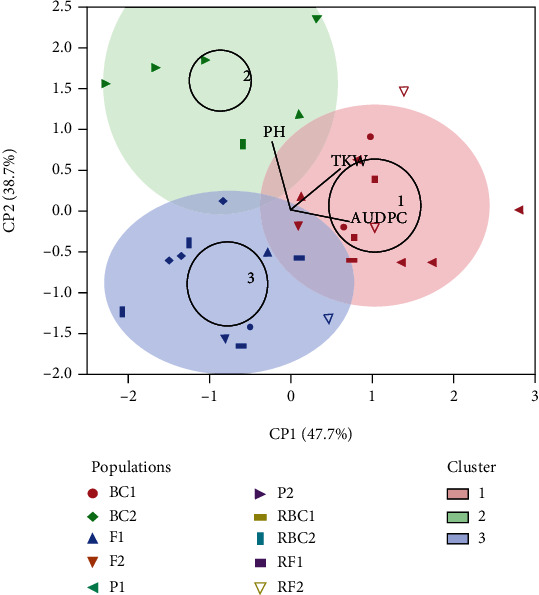
Clustering of 113 wheat genotypes representing the contribution of three factors: plant height (PH), thousand kernel weight (TKW), and the area under disease progression curve (AUDPC).

In an attempt to classify the different populations studied, PCA was performed on means of PH, TKW, and AUDPC data (Figure 6). The two first dimensions explained 86.4% of variation: dimension 1 accounted for 47.7% and dimension 2 for 38.7% of data variance. The first component (CP1) was positively correlated with AUDPC. The second component (CP2) was positively correlated with PH. The analysis showed three different classes of populations (Figure 6). The first cluster (red) contained six genotypes: P_1_, BC_1_, RBC_1_, F_1_, RF_1_, and F_2_, located on the two sides of CP1 and on the right side of CP2, with positive values of AUDPC (range 0.2–2.7) and varied values of PH (−0.6 to 1.4). Cluster 2 (green) included only four populations (P_2_, RBC_2_, F_1_, and RF_2_) located above CP1 and to the left of CP2. This group had negative values of AUDPC (−2.5 to −0.5) and positive values of PH (0.8–2.4). The last cluster (blue) contained seven generations: BC_1_, RBC_1_, BC_2_, RBC_2_, F_1_, F_2_, and RF_2_. These had negative values of AUDPC and PH and were located below CP1 (−2.2 to −0.3) and to the left of CP2 (−1.8 to−0.4).

## 4. Discussion

Yield loss and instability of disease resistance in wheat are currently mainly due to climate change. Managing wheat diseases by introducing new, effective, and diverse resistance genes into cultivars represents an important approach for a sustainable wheat production and a good alternative to fungicide treatments [8, 64, 65]. Therefore, wheat breeding programs have been improved to release high-yielding genotypes that are also resistant to major diseases [66, 67]. The breeding for disease resistance in wheat is considered one of the best, durable, economic, and environmentally friendly strategies to control biotic stresses including STB [68]. Screening wheat disease-resistant progenies within segregating populations is a major step for detecting new resistance genes against *Z. tritici* and developing practical wheat breeding programs against STB [8]. As part of this effort, investigating the quantitative inheritance of disease resistance is also needed in order to explore the possible genetic effect and the presence of reciprocal effects. Existence of this reciprocal effect in wheat resistance to *Z. tritici* and the importance of cytoplasmic genetic information as a new source of resistance could be a real advantage in field conditions [39, 69]. The introgression of *Z. tritici* resistance genes with additional cytoplasmic genetic resistance and the estimation of its heritability are of great importance to optimize conventional and molecular wheat breeding programs [64, 68]. In addition, better understanding of the relationship between nuclear and organellar genomes is also needed for the efficient use of cytoplasmic resistance [39]. Consequently, the aim of the present study was to determine the heritability, the nature of gene action, and any maternal and cytoplasmic effects governing inheritance of resistance to *Z. tritici* in durum wheat. The *Z. tritici* resistant variety Maâli revealed a significant cytoplasmic effect on STB severity using several segregating populations across three growing seasons. The analysis of variation of STB disease severity during the three growing seasons revealed significant differences in the AUDPC value between the segregating populations. Furthermore, the distribution of STB resistance in different generations of the segregating populations was correlated with the susceptibility of the female parent. Similar observations were made by Mazouz et al. [39] and Jlibene et al. [69], illustrating the genetic complexity of wheat resistance to STB disease. The disease susceptibility of the different generations also varied between cropping seasons due to environmental differences and differences in annual disease pressure. As shown by Ferjaoui et al. [25], the varieties Karim and Maâli exhibited different levels of susceptibility to STB disease, at the same experimental station of Oued Beja in two seasons (2009 and 2010). In addition, significant negative correlations of AUDPC values with TKW and PH were observed in the different populations for each growing season. This correlation between infection parameters and agronomic traits was also reported by Ramdani et al. [70] and Berraies et al. [52], where lowest yields were associated with highest STB infection levels in Morocco and Tunisia. In fact, *Z. tritici* was shown to reduce the intercepted radiation and remove soluble assimilates from the colonized host [49]. Arraiano and Brown [27] and Robert et al. [71] also indicated the influence of plant traits and crop architecture on epidemic progress. Similar results showed that PH was strongly associated with reduced AUDPC values in wheat mixtures, leading to the conclusion that vertical progress of STB disease was affected by the distance between consecutive leaves [48]. Furthermore, in the present study, the plants with the greatest height were also the most resistant to *Z. tritici,* suggesting that PH might be closely linked with the STB resistance gene in the parent Maâli. The TKW of the susceptible progenies was on average closer to the TKW of the susceptible genotype Karim. A similar result was reported by Berraies et al.[52] with 800 experimental elite durum wheat breeding lines screened for reaction to STB under natural infection at the CRRGCB experimental station, showing that the TKW trait was typically related to the *Z. tritici* susceptible parents. The PCA of the three traits of PH, TKW, and AUDPC allowed us to identify three clusters of generations. This analysis method is in agreement with the work of Hassine et al. [72] that reported the same classification of the different commercial varieties according to these important different traits.

The analysis of disease severity segregation within the populations rejected both the additive-dominance and digenic epistatic models. Thus, the epistatic model with maternal and cytoplasmic effect was applied and revealed to be adequate for studying these different populations, indicating the complexity of inheritance of resistance to *Z. tritici* compared to the additive-dominance model. Similarly, several studies reported the presence of cytoplasmic and/or maternal effects in the inheritance of many quantitative traits such as resistance to yellow berry and *Z. tritici* in durum wheat [35, 61, 73]. Mazouz et al. [39] studied the segregation pattern of the resistance to *Z. tritici* in RBC_2_ and F_2_ populations, generated from resistance bread wheat parents (THORNBIRD or RPB709.71/COC), and showed the presence of a cytoplasmic effect and the importance of the maternal effect in the disease resistance. In addition, the susceptible progenies from the reciprocal crosses what were the most susceptible to *Z. tritici* were also lacking the cytoplasm of the resistant parent [39]. The presence of an epistatic effect in resistance to *Z. tritici* was also reported in two durum wheat crosses under controlled conditions by Bnejdi et al. [35]. Similarly, Zhang et al. [74] showed that additive, dominance, and epistatic effects contributed to the expression of STB disease resistance. In addition, the negative scores of additive, dominance, and cytoplasmic effects indicated that these effects had higher contributions to disease resistance than to disease susceptibility. This result was consistent with that of Mohammadi et al.[75] who showed that additive and dominance effects have roles in controlling all traits in bread wheat such as AUDPC, percentage of necrotic leaf area, and percentage of pycnidial coverage. Different results were reported by Ramezanpour et al. [37] and Vakili Bastam et al. [38], who observed the role of partial dominance with additive gene effect on the control of inheritance of STB resistance in bread wheat.

Moreover, the absence of dominance × dominance and additive × dominance effects in this study could motivate the development of homozygous lines rather than hybrid varieties. In fact, hybrids could compromise the cross-pollination by anther extrusion, leading to a decrease in seed production, as described by Muqaddasi et al.[76]. In addition, the positive score of the additive × additive effect also suggested that the gene pairs could be in complementary form in parents, based on Mather and Jinks [77]. The same additive × additive, dominance, and epistatic effects were also found for resistance to *Z. tritici* in hexaploid wheat crosses [69]; moreover, the progeny derived from the resistant female showed a higher level of resistance. Based on crosses between six bread wheat genotypes, Mazouz et al. [39] also concluded the presence of cytoplasmic and nucleus gene effects in inheritance of resistance to *Z. tritici* by the female parent. Similarly, other studies showed the importance of maternal genetic effects in the inheritance of fungal disease resistance in several crops, such as in oat (*Avena* sp.) to *Puccinia coronata* Cda. f. sp. *avenae* [61].

Nevertheless, the high heritability and the variation of genetic effects between populations and years in our study indicate that introgression of genes governing inheritance of resistance to *Z. tritici* could be possible through conventional backcrosses. A higher heritability was also observed by Berraies et al. [7] in recombinant in bred durum wheat lines segregating for resistance to *Z. tritici*. However, the variation in STB resistance, reported by Arraiano and Brown [27], showed that phenotypic selection by crossing well-adapted cultivars from different lineages could lead to a transgressive segregation and help to breed for potentially durable polygenic quantitative resistance traits. Different methods could assist breeders to construct effective gene pyramiding in order to incorporate STB resistance into new cultivars [44, 78]. Previous studies on Tunisian durum wheat landraces showed that pyramiding resistance may potentially lead to a durable resistance [25, 79]. Therefore, phenotyping and molecular strategy are important components for the sustainability of wheat production by introducing new, effective, and diverse resistance genes into cultivars [44, 65]. Consequently, breeders need to estimate at each cycle the part of disease resistance due to nonadditive effects (dominance and epistatic components); these nonadditive effects could be successfully utilized to enhance the overall resistance level and improve breeding programs.

## 5. Conclusion

The present study indicated the presence of cytoplasmic and maternal effects in inheritance of wheat resistance to *Z. tritici*. These results suggest that the choice of female resistant parent would greatly contribute to increase the resistance to STB disease in durum wheat breeding programs. In addition, the presence of additive × maternal and dominance × maternal interactions indicated the need for investigating the relationship between nuclear and cytoplasmic–mitochondrial genes in order to efficiently take advantage of the cytoplasmic and maternal resistances. This investigation should encourage geneticists and breeders to transfer these types of resistance to elite genotypes in combination with management practices in order to reach durable STB disease control.

## Figures and Tables

**Table 1:** *Zymoseptoria tritici* isolates used in the study.

No	Origin	Province/country
1	Isolated from Karim in 2013–2014 cropping season	OuedBeja/Tunisia
2	Isolated from Razzak in2013–2014 cropping season	OuedBeja/Tunisia
3	Isolated from Maâli in 2013–2014 cropping season	OuedBeja/Tunisia
4	Isolated from Salim in2013–2014 cropping season	OuedBeja/Tunisia
5	Isolated from Karim in 2014–2015 cropping season	OuedBeja/Tunisia
6	Isolated from Razzak in 2014–2015 cropping season	OuedBeja/Tunisia
7	Isolated from Maâli in 2014–2015 cropping season	OuedBeja/Tunisia
8	Isolated from Salim in 2014–2015 cropping season	OuedBeja/Tunisia

**Table 2:** Rating scale for wheat disease assessment [54].

Scale score	Percentage of infected of leaf area (%)
1	0
2	2.5
3	10
4	25
5	50
6	75
7	90
8	97.5
9	100

**(a):** 

AUDPC		[a]	[d]	[c]	[am]	[dm]	[a × a]	[d × d]	Block	Lack − of − fit
2015	Estimate	12.1	4.7	2.9	2.85	4.32	3.39	—	3.34	4.53
	*F* value	206.53	0.66	62.85	17.39	0.22	—	—	0.08	107.5
	*P* value	<0.0001	0.4566	0.0047	0.0278	0.6589	<0.0001		0.7923	0.0003
2016	Estimate	6.97	5.8	8.46	8.72	11.5	—	3.98	5.13	5.82
	*F* value	649.5	29.02	94.94	13.2	14.43	—	35.48	0.38	58.3
	*P* value	<0.0001	0.0019	<0.0001	0.0057	0.0027		0.0040	0.5627	0.0003
2017	Estimate	11.4	8.37	6.36	7	6.56	6.19	—	6.57	5.93
	*F* value	44.01	0.12	1.23	26.63	26.13	32.03	—	0.75	145.77
	*P* value	<0.0001	0.7409	0.3079	0.0013	0.0017	0.0012		0.41	<.0001

**(b):** 

TKW		[a]	[d]	[c]	[am]	[dm]	[d × a]	[d × d]	Block	Lack − of − fit
2015	Estimate	6.08	6.14	6.66	6.74	6.05	—	—	8.41	6.31
	*F* value	0.26	6.52	2.46	3.00	2.93	—	—	1.93	36.00
	*P* value	0.6297	0.0424	0.163	0.128	0.1374			0.2003	0.0004
2016	Estimate	—	8.59	—	26.4	44.7	7.15	103	6.64	6.21
	*F* value	—	0.00	—	0.21	0.01	0.47	0.01	1.34	1.28
	*P* value	—	0.9676	—	0.6522	0.9247	0.5148	0.937	0.2862	0.3426
2017	Estimate	8.98	6.98	—		7.08			8.48	7.79
	*F* value	0.00	10.29	—	—	22.73	—	—	0.25	3.70
	*P* value	0.9896	0.0150	—		0.0020			0.6293	0.0561

**(c):** 

PH		[a]	[d]	[c]	[am]	[dm]	Block	Lack − of − fit
2015	Estimate	6.31	6.04	6.8	6.76	—	7.87	3
	*F* value	89.95	15.51	8.26	0.00	—	0.00	9.16
	*P* value	<0.0001	0.0075	0.0246	0.9715		0.9919	0.0097
2016	Estimate	8.09	6.33	6.65	6.51	—	7.5	7.1
	*F* value	63.17	29.84	2.42	5.63	—	0.20	33.63
	*P* value	<0.0001	0.0013	0.1661	0.0521		0.6648	0.0001
2017	Estimate	8.55	7.44	8.04	—	7.53	8.79	8.04
	*F* value	243.5	0.08	0.21	—	4.31	0.44	0.21
	*P* value	<0.0001	0.7800	0.6595		0.0737	0.5250	0.6625

**Table 4:** Estimates of additive, dominance, and environmental variances with ±SE (×10), narrow-sense heritability (*h*^2^*n*), and minimum number (*N*) of genes (or effective factors) for resistance to *Zymoseptoria tritici*, PH, and TKW in durum wheat crosses of Maâli (resistant parent) by Karim (susceptible parent) for three growing years.

PVC	2015			2016			2017		
	AUDPC	PH	TKW	AUDPC	PH	TKW	AUDPC	PH	TKW
VE	0.10 ± 0.01^∗^	2.16 ± 0.36	7.23 ± 2.1	0.15 ± 0.01	14.40 ± 2.4	15.1 ± 2.6	0.3 ± 0.02	5.42 ± 1.15	3.5 ± 0.20
VA^∗^	0.20 ± 0.12^∗^	23.51 ± 11.51	4.20 ± 1.2	0.45 ± 0.02	7.89 ± 1.5	7.2 ± 1.3	0.5 ± 0.01	12.11 ± 2.3	1.5 ± 0.10
VD^∗^	0.01 ± 0.001^∗^	8.13 ± 6.83	2.0 ± 0.1	0.24 ± 0.01	4.01 ± 0.2	2.3 ± 0.3	0.02 ± 0.19	0.3 ± 0.1	0.5 ± 0.01
*X* ^2^ (df = 7)	ns	ns	ns	ns	ns	ns	ns	ns	ns
*h* ^2^ *n* (%)	65	69	31	53	30	29	50	68	28
*N*	2	5	6	1.7	4	5	2.3	5	4

## Data Availability

Data are available on request.
